# Dissociable processes underlying decisions in the Iowa Gambling Task: a new integrative framework

**DOI:** 10.1186/1744-9081-5-1

**Published:** 2009-01-02

**Authors:** Andrea Stocco, Danilo Fum, Antonio Napoli

**Affiliations:** 1 Department of Psychology, Carnegie Mellon University, 5000 Forbes Avenue, Pittsburgh PA 15213, USA; 2 Dipartimento di Psicologia, Università degli Studi di Trieste, via S. Anastasio 12, 34134 Trieste, Italy

## Abstract

**Background:**

The Iowa Gambling Task (IGT) is a common paradigm used to study the interactions between emotions and decision making, yet little consensus exists on the cognitive process determining participants' decisions, what affects them, and how these processes interact with each other. A novel conceptual framework is proposed according to which behavior in the IGT reflects a balance between two dissociable processes; a cognitively demanding process that tracks each option's long-term payoff, and a lower-level, automatic process that is primarily sensitive to loss frequency and magnitude.

**Methods:**

A behavioral experiment was carried out with a modified version of IGT. In this modified version, participants went through an additional phase of interaction, designed to measure performance without further learning, in which no feedback on individual decisions was given. A secondary distractor task was presented in either the first or the second phase of the experiment. Behavioral measures of performance tracking both payoff and frequency sensitivity in choices were collected throughout the experiment.

**Results:**

Consistent with our framework, the results confirmed that: (a) the two competing cognitive processes can be dissociated; (b) that learning from decision outcomes requires central cognitive resources to estimate long-term payoff; and (c) that the decision phase itself can be carried out during an interfering task once learning has occurred.

**Conclusion:**

The experimental results support our novel description of the cognitive processes underlying performance in the Iowa Gambling Task. They also suggest that patients' impairments in this and other gambling paradigms can originate from a number of different causes, including a failure in allocating resources among cognitive strategies. This latter interpretation might be particularly useful in explaining the impairments of patients with ventromedial prefrontal cortex lesions and, by extension, the contribution of this brain region to human decision making.

## Background

Gambling paradigms are widely used to investigate the relationships between emotions and decision making in behavioral [1,2], neuropsychological [3-5], and neuroimaging studies [6-10]. The most frequently used gambling paradigm, the Iowa Gambling Task (IGT), was originally designed to measure the impulsive conduct of patients with ventromedial prefrontal cortex (VMPFC) lesions [3]. In the IGT, participants repeatedly select a card from the top of four decks, indicated as *A*, *B*, *C*, and *D*. Each selection returns an immediate win, with the amount dependent on the chosen deck. At times, however, an unexpected loss may follow the win. Losses are unpredictable, but they are scheduled so that choosing from the decks which return high immediate winnings (*A *and *B*: the "bad decks") leads to an eventual failure, while choosing from those associated with humbler gains (*C *and *D*: the "good decks") produces proportionately minor losses, yielding a gain in the long run.

While normal participants quickly learned to refrain from the bad decks, VMPFC patients perseverated on from selecting them, apparently oblivious of the future consequences [3,11,12]. Poor performances in the IGT were later reported in patients with damage in other brain regions, for example the amygdala [13], the dorsolateral prefrontal cortex (DLPFC) [14,15], and the striatum [16,17]. The IGT has proven to be sensible to a large number of impairments and a large number of psychiatric populations score significantly lower than controls, probably because they share the same decision making impairments. These include patients with schizophrenia [18-21], obsessive-compulsive disorder [22,23], depression [24,25], and alcohol or drug abuse [26-28].

### Controversies surrounding the IGT

Results from the IGT have provided the basis for a general theory of the relationship between cognition and emotion, i.e., the Somatic Marker Hypothesis [29,30]. However, although instrumental in describing the patients' behavioral impairments, this task has an unstructured nature that poses limitations in characterizing the cognitive processes underlying it [31]. This lack of agreement has lead to a number of disputes in the literature.

Theories have differed, for example, on their proposed role of the VMPFC in decision making. According to some researchers, the VMPFC is primarily involved in binding somatic states with the actions that initially caused them. VMPFC damage prevents this binding, as well as the anticipatory alerting signal that comes from expecting the stored somatic states before repeating the same action [29,30,32]. Other authors have argued, however, that the VMPFC is needed to revert previously learned associations, in particular following negative feedback [33,34]. Still others have claimed that the VMPFC biases decision making by facilitating the emotional experience of regret [5,6].

Researchers also divide on whether unconscious emotional processes underlie successful decision making in the IGT. One landmark study [11] found that skin conductance responses, which characteristically foretell selections from bad decks [35], appeared before participants were able to explicitly verbalize their decision strategies. Participants' awareness, however, was only assessed with open-ended, verbal questions, a method that lacks sufficient sensitivity to detect all the relevant task knowledge (e.g., [36]). When a more structured, written questionnaire was adopted [37], or when participants were simply asked to rate each deck's goodness [18,38], the results suggested participants knew more about the task than previously believed. Furthermore, this knowledge was correlated with successful performance of the task [37]. Still, the presence of explicit knowledge does not necessarily rule out implicit components. When participants' knowledge is assessed by means of indirect, behavioral techniques, they do exhibit evidence that their decisions are at least partly driven by unconscious decision biases [39]. In summary, participants' explicit knowledge was initially underestimated, and is likely to be the main force driving their behavior. However, processes that are automatic and implicit in nature do exert subtle influences in orienting decisions, and their effects can be traced in form of decision biases.

A third, important debate concerns whether emotional decision making can be carried out independently of general cognitive resources, and, in particular, of working memory. For example, one study [40] contrasted the performance of VMPFC patients against patients with DLPFC lesions. All patients performed the IGT and two working memory tasks. The results indicated a double dissociation, with VMPFC patients impaired in the IGT but not in the two working memory tasks, and DLPFC patients showing the opposite pattern. Subsequent research, however, failed to replicate these findings. Two studies reported that DLPFC damage negatively affects IGT performance [14,15], and one found a correlation between working memory impairment and low performance on the IGT in individuals with substance dependence [41]. Experiments with healthy controls also produced contrasting results. Turnbull and colleagues [42] compared the performance of three groups of participants in the IGT. The first group served as a control. The second performed the IGT together with a concurrent, shallow task that did not tap into central cognitive resources (i.e., verbally repeating the sequence of numbers 1 to 9). The last group performed a concurrent task that is known to load working memory and central resources (i.e., generating random numbers). Surprisingly, performance was comparable across all three groups, suggesting that successful decision making in the IGT depends on rather automatic processes that do not rely on central resources. A number of other experiments, however, adopted similar design and found different results. In particular, they reported that IGT performance was consistently lower when the interfering task was demanding for working memory than when it was not [43-46].

Without a clear understanding of the cognitive processes recruited by the IGT, it is difficult to make sense of these results. It is entirely possible, for instance, that working memory resources are needed only at *certain *moments during the task. For instance, participants might simply stick with their initially preferred decks until losses become noticeable. Then, they might pause and start to reason about each decks' outcomes to reconsider their profitability. When done with this assessment, they can simply proceed with their new preferred options for another while. This possibility is explicitly incorporated in the framework presented in this paper. Importantly, this possibility makes it difficult to evaluate a secondary task's effectiveness, if the task is self-paced and its performance is not checked (as in [42]). An ideal interfering task, therefore, should exert its cognitive toll consistently throughout the IGT. In fact, *any *manipulation that consistently prevents participants from devoting time and cognitive resources for an accurate deck assessment should negatively impact decisions making. A recent paper [47] seems to confirm this intuition. In this study, the authors found that IGT performance was impaired when participants had to select a card within two seconds. Such a severe time constraint might have served the purpose of reducing the time necessary to adequately evaluate the decks, even in absence of any interfering task.

### Additional limitations of the IGT

For the purposes of this paper, two additional limitations are worth mentioning. The first is that performance in the IGT has been traditionally analyzed in terms of long-term payoff. This fact betrays the assumption that emotional decision making obeys the classical economic theory in which the decision maker maximizes the expected values of options. In contrast with this assumption, some studies have reported a singularity in the distribution of choices: deck *B *(one of the two disadvantageous decks) is usually chosen more frequently than each of the two advantageous decks taken separately [48,49]. Other studies also consistently show that participants are sensitive to the frequency of losses, and that, when payoff is kept equal, they tend to select from those decks where losses are less frequent [45,50-52].

In fact, experimental evidence suggests that losses influence human decision making more than the simple payoff. Participants typically reject gambles that offer equal probabilities of gaining and losing money [53,54], and they value an object they have to part from more than they would pay for it [55-57]. Moreover, prospect theory [58], the most influential behavioral model of human decision making, explicitly assumes a value function where losses are perceived as greater than gains of equal amount.

There is also evidence that the effect of monetary losses is modulated by their frequency and temporal proximity. For example, participants are more likely to reject a risky gamble if their previous bet resulted in a loss than if it resulted in win, even if the two gambles are totally independent [2]. In the brain, neuroimaging studies have shown that reward-related activation in the prefrontal cortices is temporally discounted, so that rewards or losses distant in time elicit weaker responses than closer ones [59]. A temporal discount enhances the sensitivity to frequency, since smaller but closer losses weigh more than larger, remote ones. In computational models of reinforcement learning, loss frequency affects action selection [60]. These types of algorithms constitute reliable models of the basic reinforcement signals in the brain [61] and have been used to model participants' choices in the IGT [62].

A second, noteworthy limitation derives from the iterative nature of the IGT. In this task, participants are continuously interleaving two kinds of cognitive operations: learning about the task structure from the cards' feedback, and using this information to decide which deck to select from. Traditionally, patients' abnormal behavior has been imputed to the decision component [3,12]. It is equally possible, however, that patients' impairment originates from an impasse in the learning stage, or a combination of the two. An experiment by Fellows and Farah [33] provides evidence in this sense. The authors tested a group of VMPFC patients with a modified version of the IGT, where deck losses were presented immediately, in the very first few choices. When patients had the opportunity to evaluate losses upfront, their performance reverted to normal. This finding suggests that their specific disablement was due more to relearning the correct associations between deck and payoff after they were initially established than to faulty decision making. It is clear, therefore, that interpretation of patients' failures within the IGT crucially depends on the distinction between learning and the decision stages. At the best of our knowledge, this distinction has been overlooked in the literature thus far.

To summarize, the IGT is a popular and valuable research paradigm that taps into the interaction between emotion and cognition. However, a deeper understanding of the cognitive processes underlying decisions in the task is needed to make sense of a number of issues. These include: (a) the role of implicit decision biases, possibly of affective nature; (b) the role of working memory in assessing each deck's value; (c) participants' biases towards low-frequency losses; and (d) the interplay between learning from decision outcomes and applying this acquired knowledge to the decision process.

### An integrative framework

In order to investigate the possible processes underlying participants' behavior in the IGT, one needs a preliminary characterization of how these processes outlined above contribute to decision making, what affects them, and how they interact. In this paper, we outline and test a simple organizing framework based on three assumptions.

The first assumption is that participants' behavior is driven by at least two competing forces; the tendency to choose a deck based on its perceived ultimate payoff and the tendency to select a deck based on previous emotional reactions to experienced wins and losses. This second tendency can be affected by a number of different factors. The most obvious one is the perceived frequency of losses and wins among decks. While other factors might play a role (e.g., the outcome magnitude, although in previous experiments [50] we found that this feature did not play a causal role in determining the participant's choices), we will consider the frequency of losses and wins as the primary force driving decision making based on emotions.

The second assumption is that the cognitive processes mediating these tendencies are independent, and potentially underpinned by different brain circuits. This assumption originates from the fact that detecting long-term payoff requires higher-level cognitive abilities and working memory to keep track of wins and losses. It also implies that payoff estimation is by nature a more difficult task. Therefore, this process is more likely to fail and select the wrong decks – especially when cognitive resources are depleted, or deck payoffs are made harder to discriminate. Emotional reactions to decision outcomes, on the other hand, are widely regarded as an automatic and less demanding process.

The third assumption is that behavior is guided by the representations produced by these two processes while learning from the decision outcomes. For instance, participants might construct and revise the estimates for the mean gain of a deck while evaluating the long-term payoff of an option, and concurrently form somatic memories of their emotional reactions when a win or a loss is unveiled. These two types of representations differ in richness, articulation, and easiness of access with payoff-sensitive representations being more explicit and difficult to create, and emotional memories being closer to intuition-mediated processes in decision making. One important consequence of this assumption is that, if learning is suddenly interrupted, participants can still base their decisions on representations they have previously acquired through either process.

### Experimental predictions

Although simple and qualitative, this framework allows a number of hitherto untested predictions. The first, straightforward prediction is that participants' preferences (in terms of payoffs or loss frequency) must be learned during the IGT. That is, under normal conditions of iterative, repeated choice preferences for loss frequency and payoff will change over time and exhibit learning curves as information is progressively gained about each deck's wins and losses.

The second prediction is that the presence of a secondary task should affect only participants' sensitivity to the decks' payoffs, and not their preferences in terms of frequency. This follows from our second assumption that the process underlying sensitivity to payoff, but not loss frequency, is cognitively demanding and requires central resources.

The third prediction is that a secondary task should affect only the learning phase, while it should not alter participants' decision behavior after learning has occurred. This follows from the third assumption that, once learning has occurred, knowledge remains accessible and can be quickly used for deciding, even in spite of an interfering task.

The fourth prediction is that monetary losses should have little or no impact on participants' preferences in terms of payoffs, but should nonetheless elicit emotional reactions that bias preferences in terms of frequency. Emotional reactions are likely to depend upon the magnitude of the loss, with larger losses resulting in stronger aversive reactions. Because of the internal constraints of the tasks, the two decks that deliver losses less frequently (*B *and *D*) also yield larger-magnitude losses than their similar-payoff counterpart (i.e., *A *and *C*, respectively). Therefore, they are more likely to be avoided immediately after experiencing an unfavorable result. This implies that choices based on loss frequency should be more likely after a positive than a negative outcome.

The third and the fourth predictions imply a double dissociation between payoff- and frequency-tracking processes. If the two were completely independent, there would be no interaction between the presence of monetary loss and the presence of an interfering task. However, the distribution of choices based on loss frequency information is likely to change when information on payoff is available. Since the acquisition of payoff information is hindered by an interfering task, a fifth prediction can be derived, stating that the presence of a secondary task should interact with the experience of a previous monetary loss in terms of frequency, but not in terms of payoff.

## Methods

To test these predictions, one needs to identify behavioral correlates of the two processes outlined above (the high-level one that orients towards payoffs and the lower-level one that tracks loss frequency). Fortunately, in most versions of the IGT, payoff and loss frequency are independently manipulated among decks. Two decks (*A *and *B*) have negative payoff, while in the remaining two (*C *and *D*) the payoff is ultimately positive. Of each pair, one deck (*A *and *C*) has a high frequency of losses (5 losses out of every 10 trials), while in the other (*B *and *D*, respectively) losses appear with low frequency (1 loss out of every 10 trials). Therefore, behavioral preferences in terms of payoff and frequency can be tracked by grouping participants' choices according to these two criteria. We will refer to these two measures of performance as the *P *(for payoff) and the *Q *(for frequency) indexes.

A second requirement is to have some means of separating the learning and the decision phases. Stocco and Fum [39] solved this problem by having participants perform an additional "blind" phase after a standard initial learning phase with the IGT. During this phase, participants were invited to continue selecting as previously, but wins and losses were kept secret, and the net amount of money won was only revealed at the end. Since learning cannot occur in this phase, behavior depends entirely on the decision component, and a comparison with the previous phase permits an estimation of the learning component. The experiment presented in this paper intentionally exploits this manipulation to test the framework's predictions. In the experiment, participants initially performed a standard, 100-choice interaction with the IGT. When this phase was completed, they performed a second, 20-choice blind phase, allowing us to measure decision performance uncontaminated by further learning.

A secondary, distractor task was introduced during either the initial learning phase or during the additional blind phase to estimate the contribution of central cognitive resources when learning is involved (first phase) or not (second phase). Therefore, three factors were manipulated: the Phase (first vs. second, within subjects), the Condition in the first phase (Dual task vs. IGT only, between subjects) and the Condition in the second phase (Dual vs. IGT, between subjects). During both phases, participants' performance was assessed both in terms of payoff and loss frequency.

### Participants

One-hundred fifty-two unpaid volunteers (mean age 22.2, SD = 2.9, 81 females) were recruited at the University of Trieste, Italy. Since age [64] and education [65] seem to affect IGT performance, participants were included only if their age fell inside the range of 18–34 years (corresponding to the young adults group in [64]) and were enrolled in a first-level college degree ("Laurea Triennale", corresponding to 13–15 years of education).

Each participant was randomly assigned to one of the four groups (Dual/Dual, Dual/IGT, IGT/IGT, and IGT/Dual). Paired comparisons ensured that the four groups did not differ from each other on either age (*p *> .05, Wilcoxon test, uncorrected) or gender distribution (*p *> .30, χ^2 ^test, uncorrected).

### The interfering task

The interfering task was designed to be fast-paced and consistently cognitive demanding. Participants were auditorally presented with a series of numbers in the range 1–10. For each number, they had to indicate whether it was even or odd by pressing one of two buttons on a numeric keypad. To make sure the secondary task did not interfere with the main task at the irrelevant level of perceptual/motor abilities, numbers were presented auditorally and the answer was given with the non-dominant hand. The lag between the onsets of two consecutive stimuli was fixed to two or three seconds. Stimuli were presented in random order, with the only constraint that the same stimulus could not occur twice in a row.

### Procedure

Experimental sessions were held individually. Participants received written instructions, which were an Italian translation of those in [12]. After reading the instructions, they completed the first phase of a computer version of the IGT. The software used was a custom-made replica of the program originally used in [12]. Decks were visually presented in the lower part of a 15 in. LCD screen, and participants used a mouse device to point and select the deck they had chosen. Immediately after each card selection, the amount of money won (and possibly lost) was displayed in the center of the upper half of the screen. The presentation of wins and losses lasted 6 s, during which the decks were grayed out and no card could be selected. The running total of money was always visible in the uppermost part of the screen and was updated after each selection.

Before the second phase, the experimenter gave new written instructions to participants and ascertained their comprehension. These new instructions emphasized that decks' profitableness was unchanged, and participants could continue selecting as they did before. No wins or losses were presented after any card selection during this phase, but the decks were still grayed out for the same amount of time to keep the interaction consistent with the previous phase. The IGT was performed in the *A'B'C'D' *version [12]. This version was chosen because, in our previous experiments and pilot studies, it had produced an evener distribution of choices between the two low-frequency decks (*B *and *D*).

In the secondary task, stimuli were presented through a pair of wireless earphones, and participants responded by pressing one of two keys on a numeric pad. Participants were instructed to use their non-dominant hand to press the two keys, leaving their dominant hand free to use the mouse for the main task. This way, the motor interference between the two tasks was minimized. Additionally, all the participants underwent a brief training session with the secondary task procedure before the beginning of the experimental session where it was adopted.

### Data analysis

The most common performance measure in the IGT is the difference between the number of good (*C *and *D*) and bad (*A *and *B*) decks selected in a series of consecutive choices. This is a measure of payoff, and, for convenience, we adopted it as a proxy for *P*:

*P *= (*C *+ *D*) - (*A *+ *B*)

To obtain a comparable measure for participants' sensitivity to *frequency*, *Q *was similarly calculated as the difference between the number of choices from the low- (*B *and *D*) and the high- (*A *and *C*) loss-frequency decks:

*Q *= (*B *+ *D*) - (*A *+ *C*)

## Results and discussion

To assess compliance with the instructions and the effectiveness of our design, we excluded those participants who, in the secondary task, responded to less than 60% of the stimuli (in the 3 s condition), or 40% (in the 2 s condition) in either phase. Three participants fell below this criterion, and their data were therefore removed from all subsequent analyses.

A preliminary series of tests was then run on the secondary task data. Table 1 reports the mean accuracy rates for both phases under the two lag conditions. Accuracy was analyzed separately for the two phases, using a mixed-effects ANOVA with Lag (2 s vs. 3 s) and Group (Dual/Dual vs. Dual/IGT for Phase 1, and Dual/Dual vs. IGT/Dual for Phase 2) as factors. In these as well as in all the following analyses, subjects were the random factor. Accuracy rates were arcsine-root transformed before being submitted to the analysis. In both phases, only the Lag factor was significant [Phase 1: *F*(1, 74) = 60.82, *p *< .0001; Phase 2: *F*(1, 73) = 6.64, *p *= .01; all other factors or interactions: *F *< 1.62]. In particular, participants responded less accurately in the 2 s then in the 3 s condition (see Table 1).

**Table 1:** Performance in the secondary task.

		Phase	
		First	Second
Lag	2 s	0.86 ± 0.08	0.93 ± 0.05
	
	3 s	0.95 ± 0.03	0.96 ± 0.05

Mean accuracy (± SD) for the Secondary Task, divided by Phase and Lag duration.

A second series of analyses was conducted to uncover any effect of Lag factor on IGT performance. Separate ANOVAs were conducted for the two phases, using Lag and Group as factors, and either *P *or *Q *as the dependent measures. The Lag had a significant effect only on the *Q *index in the second phase [*F*(1, 73) = 4.78, *p *= .03], with participants drawing more often from the low-frequency decks in the 2 s (*Q *= 13.46, *SD *= 5.98) than in the 3 s condition (*Q *= 10.86, *SD *= 4.38). This effect could be due to the lack of feedback in this phase; unaware of their performance, the increased pressure of the task could have pushed participants to rely more than usual on the shallow, emotion-driven decision process. All the other factors or interactions were non-significant (*F *< 3.15). Because the Lag factor did not have important effects on IGT performance, and in order to increase statistical power, data were collapsed across Lag conditions in all the remaining analysis.

In order to test our experimental predictions, the values of *P *and *Q *were calculated for each participant for each consecutive block of 20 choices in the first phase. Analyses of variance were then performed on *P *and *Q *separately, using the Block (5 values, 01–20 to 81–100), the Condition in the first phase (2 values, Dual vs. IGT), and the Condition in the second phase (2 values, Dual vs. IGT) and as factors. The latter was included to make sure that the experimental groups did not differ already in the first phase, while the Block factor was included to capture the learning curve.

**Figure 1:**
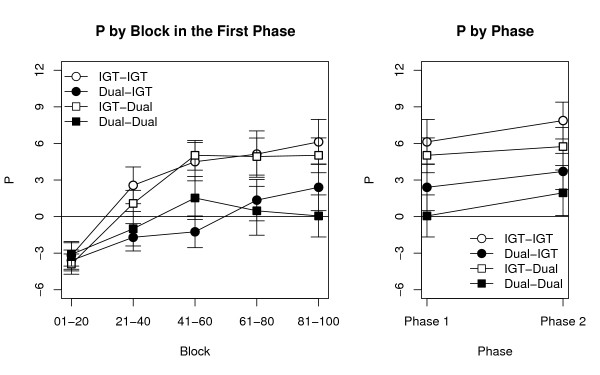
**The *P *index across the two phases**. Time course of the *P *index (tracking the preference for decks with high payoff) for the four groups during the experiment. **Left: **values of *P *during the five blocks of the first phase, grouped by Condition in the first and in the second phase. **Right**: A comparison of *P *at the end of the first phase and during the second phase, where no feedback was allowed. Points represent mean values ± SEM.

**Figure 2:**
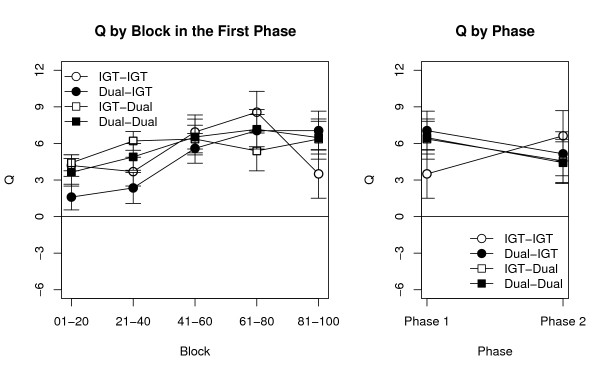
**The *Q *index across the two phases**. Time course of the *Q *index (tracking the preference for decks with low frequency of losses) for the four groups during the experiment. **Left**: values of *Q *during the five blocks of the first phase. **Right**: A comparison of *Q *at the end of the first phase and during the second phase, where no feedback was allowed. Points represent mean values ± SEM.

The left panels in Figures 1 and 2 illustrated the time courses of *P *and *Q*, respectively, plotted through each 20-choice block for the four groups. As expected, the Condition in the second phase did not have a significant effect on either *P *[*F*(1,145) = 0.04] or *Q *[*F*(1,145) = 0.62], ensuring that our experimental groups did not differ already in the first phase. For participants who did not perform a secondary task, the time course and the mean values for *P *were comparable with those previously reported for groups of similar age and education [39,47,64,65].

Confirming our first prediction, the main effect of Block was significant for both *P *[*F*(4, 580) = 23.84, *p *< .0001] and *Q *[*F*(4, 580) = 7.46, *p *< .0001]. The Condition in the first phase, however, was significant only for *P *[*F*(1, 145) = 9.52, *p *= .002] while did not affect *Q *[*F*(1,145) = 0.16]. This asymmetry confirmed our second prediction, and implies that processes underlying payoff preference are more dependent on central resources than those underlying frequency preference. This is indicated by the clear separation between the two Dual (in black) and the two IGT groups (in white) in the left panel of Figure 1, in contrast to their comparable trajectories in the left panel of Figure 2. A predictable interaction was found between Block and the Condition in the first phase in the case of *P*; participants in the Dual condition exhibited a slower learning curve [*F*(4, 580) = 3.02, *p *= .02]. No other interaction among factors was significant for either *P *(*F *< 1.19) or *Q *(*F *< 1.81).

Testing our third prediction requires a comparison between the two phases. Since they have different lengths, participants' performance in the last block of the first phase was used as proxy for the entire phase (as in [39]). A graphic illustration of these comparisons for *P *and *Q *is provided in the two right panels of Figures 1 and 2, respectively. Two separate ANOVAs were run using Phase (2 values, First vs. Second), Condition in the first phase, and Condition in the second phase as factors, and either *P *or *Q *as dependent variables. As in the previous analysis, the Condition in the first phase had a significant impact on *P *[*F*(1, 145) = 8.06, *p*. = .005], but not on *Q *[*F*(1, 145) = 0.11]. However, the effect of Condition in the second phase was not significant for either measure [*P*: *F*(1, 145) = 1.66; *Q*: *F*(1, 145) = 0.01]. Neither the main effect of Phase nor any of the interactions were significant (*P*: *F *< 2.75; *Q*: *F *< 2.06). Therefore, performance is determined by the condition under which the first phase, but not the second, is run. These results confirm that the involvement of central cognitive resources is limited to learning and estimating the decks' payoffs; and that, when deciding which deck to select, participants rely on knowledge that is easily accessible and cannot be interfered with once it has been acquired.

**Figure 3:**
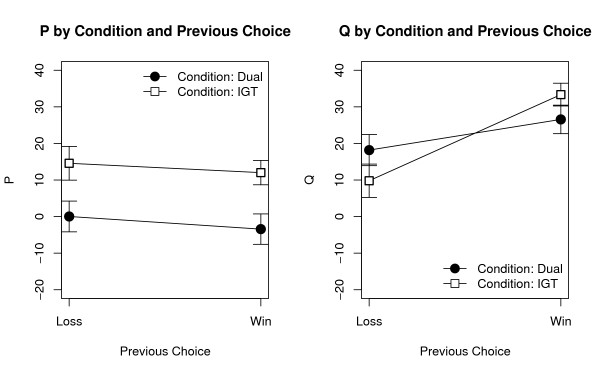
**Dissociation between *P *and *Q***. **Left: **Effects of Condition in the first phase and presence of a loss in the previous selection on *P*. Only the Condition has a significant effect on *P*. **Right: **Effects of Condition in the first phase and Presence of a Loss in the previous selection on *Q*. This time, only the Loss in the previous phase has a significant effect. Points represent mean values ± SEM.

The last two predictions concern the behavioral effects of a monetary loss. To test them, all the participants' choices in the first phase were divided in two categories: those following a selection that yielded a loss, and those following a selection that produced a win (choices in the second phase were excluded since wins and losses were kept hidden.) The mean values of *P *and *Q *were computed for each participant in each category. Two ANOVAs were then run using the Condition in the first phase (2 values, Dual vs. IGT) and the experience of a loss in the Previous selection (2 values: Loss vs. No Loss) as factors, and *P *and *Q *as the dependent measures. As expected, *P *was significantly affected by the Condition [*F*(1, 147) = 8.67, *p *= .004], with participants in the Dual condition (*P *= -1.70, *SD *= 36.64) scoring lower than those in the IGT condition (*P *= 13.29, *SD *= 33.53), but was not affected by a Previous loss [*F*(1, 147) = 1.21: see Figure 3, left]. On the contrary, *Q *was not affected by the Condition [*F*(1, 147) = 0.04], but was significantly affected by a Previous loss (*F*(1, 147) = 16.80, *p *< .0001): as predicted, participants' tendency to select from the low-frequency decks was higher after a win (*Q *= 29.80, *SD *= 30.68) than after a loss (*Q *= 14.18, *SD *= 37.78: See Figure 3, right). Confirming our fifth prediction, the two factors interacted significantly for *Q *[*F*(1, 147) = 3.96, *p *= .05], but not for *P *[*F*(1, 147) = 0.03].

As can be seen in the right panel of Figure 3, the effect of a monetary loss on *Q *was larger for those participants who *did not *perform the interfering task. This result is apparently counterintuitive, but can be explained by assuming that, after a large loss (as those produced by *B *or *D*), participants would immediately refrain from the deck just selected. When payoff information is not available, participants can distribute their choices over the remaining three decks. When information on the payoff structure is available, however, participants might redistribute their preferences among those decks with a similar perceived payoff, i.e. prefer *C *after a loss caused by *D*, and *A *after a loss caused by *D*. This does not alter the global payoff preference, but selectively harms those decks (*B *and *D*) that were previously preferred on the basis of loss frequency. Therefore, when payoff information is available, the effect of a loss on *Q *is stronger.

## Conclusion

Our experiment showed that performance on the IGT depends on the interaction between two separable cognitive components. Although we did not collect individual covariate measures of specific cognitive abilities, our experimental design permits a preliminary characterization of these two components. The first of these includes those higher-level processes involved in tracking and discovering the long-term payoff structure of the task. The second is based on the immediate emotional reactions to wins and losses, and is responsible for participants' sensitivity to loss frequencies and reactions to loss magnitude. These two components can be tracked by aggregating participants' choices according to the decks' payoff or loss frequency, respectively. In fact, our results show a double dissociation between these two processes. The first, but not the second, is harmed by the additional demands of an interfering task; and the second, but not the first, is affected by the recent experience of a monetary loss.

Additionally, our design allowed us to separate the decision stage, where participants evaluate their options and perform a selection, from the learning stage, where participants have the opportunity to learn about each deck's returns. This was achieved by having participants perform the IGT in two phases. Feedback was suspended on the second phase, so that further learning was prevented. Our results show that an interfering task disrupts performance only in the first phase, but not in the second. This shows that the learning stage, during which each decks' wins and losses are estimated, critically requires central cognitive resources. By contrast, the decision phase itself, whether driven by payoff or loss frequency, can be carried out efficiently in spite of an interfering task. As far as we know, this is the first successful attempt to separate these two stages.

Other authors have previously attempted to decompose the IGT into component processes (e.g., [11,66]). Our framework, however, advances these attempts in two directions. First, it accounts for a richer measurement of performance, based on loss frequency as well as payoff. Second, it includes a more elaborate specification of the type of knowledge required by the two processes, including a description of the conditions under which decision making requires central cognitive resources.

Our conclusions are hinged on the secondary task's disruptive effect on performance, as measured by the *P *index. In line with previous studies that adopted a dual-task paradigm to the IGT [42-45], this result was interpreted as arising from competition for central cognitive resources, such as working memory. One might argue that the task we adopted is not normally used to saturate these resources. However, as we argued in the introduction, the IGT does not require a *constant *use of working memory or other cognitive resources. Participants can use these resources intermittently during the task, and perform long streaks of selections building on either their previous estimates or their intuition. The task we designed and used is effective in that it consistently prevents participants from reverting back to the IGT for the amount of time needed to perform satisfactory payoff estimation.

An alternative explanation for our findings could be that the time constraints posed by the secondary task have changed participants' attitude towards risky options. In particular, time constraints might make them less averse to risk and more prone to select from the bad decks. In fact, a recent study has shown that brain responses that correlate with decision risk are slower and can be temporally dissociated [9] from those that correlate with processing a decision outcome [7]. If risk processing indeed takes more time, it is also more easily affected by the distractions posed by a fast-paced interfering task. Furthermore, this account could explain both our results and those obtained in [47], where decreased IGT performance was obtained by reducing the available decision time with no interfering task.

One problem with this alternative interpretation arises when we define risk. In the economic literature (e.g.,[67,68]) as well as in neuroimaging studies of decision making [7,9], risk is operationalized as the variance of returns within an option. In turn, variance is at maximum when the frequency of losses is higher. It follows that the high loss-frequency decks, *B *and *D*, should be perceived as riskier, and that any attitude change towards risk should impact the *Q*, and not the *P*, index. On the contrary, our data show that the secondary task affected *P*, but had no effect on *Q*.

Another problem with the risk-aversion hypothesis is that effect of risk aversion should be observed during the decision stage, when participants ultimately decided which deck to pick. However, our design allowed us to separate the decision stage from the learning stage within the IGT, and infer that the effects of a secondary task are limited to the learning stage. This conclusion was based on the fact that the secondary task did not alter performance in the second, blind phase – after learning has occurred. Had the secondary task altered participants' attitude towards risk, performance in the second phase should have been affected as well. Therefore, we conclude that our account is preferable to the risk-aversion interpretation of our results.

The findings reported in this paper can also contribute to interpret a number of results in the IGT literature. Importantly, they are relevant to the debate over the existence of unconscious components of decision making in the IGT. In our dual-process framework, implicit and explicit components co-exist, and jointly contribute to the behavioral choices. Identifying one or the other depends on how performance data is examined. For instance, selecting from the advantageous decks requires a demanding evaluation of each decks' payoff that can occur only under conscious control. It is therefore predictable that performance, when measured only by payoff, correlates with participants' conscious knowledge of the task [19,37]. On the other hand, the low-level process that responds to loss frequency and magnitude does not show up in this sort of measurement. In our results, we detected it in the *Q *index. It was possibly underlying participants' biases and perseverations in [39]. An interesting possibility is that anticipatory physiological reactions, such as skin conductance responses, can reflect the contribution of this low-level process to some extent. The relative independence of this process from payoff preferences could explain how physiological responses were found in anticipation of disadvantageous choices in the original version of the task [11,35], but in anticipation of advantageous choices under a different distribution of losses [69].

Even more importantly, our results might be useful for better understanding the source of impairment in clinical populations performing in the IGT. Our framework suggests that there are at least three potential sources for patients' abnormal behavior.

A first source of impairment is the inability to allocate sufficient cognitive resources to the IGT. This prevents patients from conducting a sufficiently accurate assessment of the decks, and, therefore, leads to detectable performance differences in terms of payoff measures (e.g., the *P *index) when compared with controls. This is the case, for instance, of patients with working memory impairment due to prefrontal lesions [15]. Although similar impairments are not affective in nature, they can possibly originate from an emotional disorder. In depression, for instance, mental rumination hinders executive functions and problem solving abilities [70,71], and can thus interfere with patients' efforts to examine the payoff structure of the task.

A second source of impairment is in the emotional reactions to losses. Our results show that experienced monetary losses trigger a temporary shift in decision preferences. In patients with affective disorders, this change might not take place at all (e.g., [2]), or might occur and have longer behavioral effects. Because of the structure of the IGT, this kind of impairment affects performance measured by the *Q *index, but does not necessarily impact the traditional payoff-based performance scores. Regrettably, however, most patient studies have assessed IGT performance only in terms of payoff, and might have overlooked important significant differences in this measure.

Our framework allows for a third, potential source of impairment. It states that participants have two types of knowledge, one deriving from careful payoff estimation and one from emotional reaction to losses or wins. Not only these two types of information, but also their relative utilities need to be learned – and ultimately learned from feedback on one's own actions. Therefore, we propose that affective reactions play a double role in decision making, and that they are used not only to evaluate decision options, but also for guiding meta-decisions on how to select among alternative decision policies.

Converging lines of evidence indicate that, for example, VMPFC patients' impairments could originate at this level. For instance, these patients poorly organize their search of information about decision options. In the IGT, they persist in selecting from the disadvantageous decks even when they are aware that the other options have higher returns [11]. Finally, this explanation is consistent with their inability to re-learn deck-outcome associations [33].

In fact, we suggest that decision-making impairments in patients with affective disorders often originate at this meta-strategic level. As such, they do not betray a malfunction in an isolated emotional decision making system, but problems in exploiting emotions to consolidate decision policies and organize behavior.

## Abbreviations

IGT: Iowa gambling task; VMPFC: Ventromedial prefrontal cortex; DLPFC: Dorsolateral prefrontal cortex.

## Competing interests

The authors declare that they have no competing interests.

## Authors' contributions

AS formulated the research hypothesis, designed the experiment, analyzed the data, and wrote the manuscript. DF and AN formulated the research hypothesis, designed the experiment, and wrote the manuscript.
